# Transcriptome analysis of Kunming mice responses to the bite of *Xenopsylla cheopis*

**DOI:** 10.1186/s13071-024-06331-4

**Published:** 2024-06-07

**Authors:** Xinchang Lun, Yuan Shi, Yiguan Wang, Ning Zhao, Qiyong Liu, Fengxia Meng, Xiuping Song, Jun Wang, Liang Lu

**Affiliations:** 1 National Key Laboratory of Intelligent Tracking and Forecasting for Infectious Diseases, National Institute for Communicable Disease Control and Prevention, Chinese Center for Disease Control and Prevention, Beijing, 102206 People’s Republic of China; 2 School of Public Health, Cheeloo College of Medicine, Shandong University, Jinan, 250012 Shandong People’s Republic of China

**Keywords:** Flea bites, *Xenopsylla cheopis*, RNA-seq, Transcriptome analysis, Ectoparasite–host interactions

## Abstract

**Background:**

Flea bites could trigger a series of complex molecular responses in the host. However, our understanding of the responses at the molecular level is still relatively limited. This study quantifies the changes in gene expression in mice after flea bites by RNA sequencing (RNA-seq) from their spleens, revealing the potential biological effects of host response to flea bites.

**Methods:**

RNA-seq was used for transcriptome analysis to screen for differentially expressed genes (DEGs) between the control mice group and the flea bite mice group. Gene ontology (GO) analysis and Kyoto Encyclopedia of Genes and Genomes (KEGG) analysis were performed on DEGs. Protein–protein interaction (PPI) network analysis on DEGs related to immune processes was performed. Finally, we randomly selected several genes from the screened DEGs to validate the results from the transcriptome data by real-time quantitative reverse transcription polymerase chain reaction (RT-qPCR).

**Results:**

A total of 521 DEGs were identified, including 277 upregulated and 244 downregulated. There were 258 GO terms significantly enriched by upregulated DEGs and 419 GO terms significantly enriched by downregulated DEGs. Among the upregulated DEGs, 22 GO terms were associated with immune cells (e.g., B cells and T cells) and immune regulatory processes, while among the downregulated DEGs, 58 GO terms were associated with immune cells and immune regulatory processes. Through PPI analysis, we found that CD40 molecules with significantly downregulated expression levels after flea bites may play an important role in host immune regulation. Through KEGG pathway enrichment analysis, a total of 26 significantly enriched KEGG pathways were identified. The RT-qPCR analysis results indicated that the transcriptome sequencing results were reliable.

**Conclusions:**

Through in-depth analysis of transcriptome changes in mice caused by flea bites, we revealed that flea bites could stimulate a series of biological and immunological responses in mice. These findings not only provided a deeper understanding of the impact of flea bites on the host but also provided a basis for further research on the interaction between ectoparasites and the host. We believe that digging deeper into the significance of these transcriptome changes will help reveal more about the adaptive response of the host to ectoparasites.

**Graphical Abstract:**

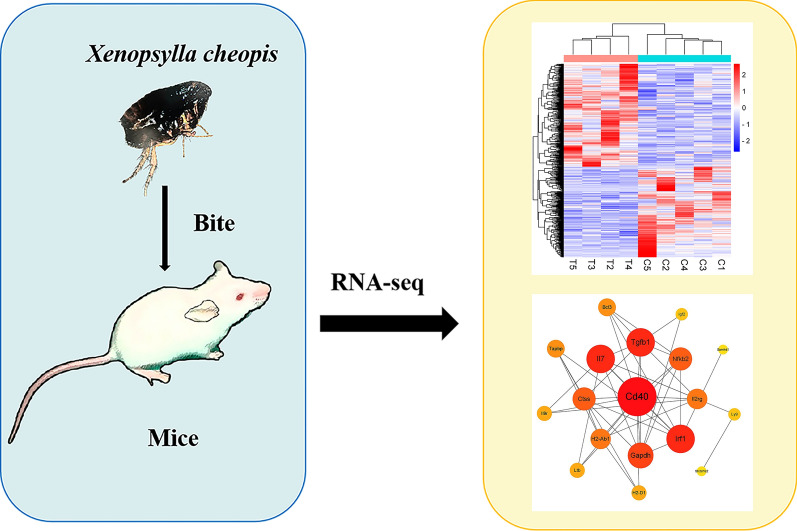

**Supplementary Information:**

The online version contains supplementary material available at 10.1186/s13071-024-06331-4.

## Background

The blood-sucking behavior of arthropods has evolved independently over 20 times [1]. The blood-sucking arthropods can regulate the defense mechanisms of the host, including their immune and hemostatic systems, through their saliva mixtures containing bioactive components, thereby preventing the occurrence of blood clotting, inhibiting platelet aggregation, and regulating the immune response of the host [1, 2]. Siphonaptera, commonly known as fleas, is an order of wingless arthropods with more than 2500 species placed in 16 families that is good at jumping [3–5]. Flea bites usually have pruritic characteristics and cause an erythematous and edematous reactions [6], leading to allergic reactions of skin lesions in the host [7]. Flea bites are not only a physical stimulus to the host but also a potential biological threat that can trigger the activation of the immune system of the host, leading to inflammatory reactions and other physiological adjustments.

*Xenopsylla cheopis*, also known as oriental rat flea, is an ectoparasite that feeds on blood and is an important arthropod vector for spreading pathogens such as *Yersinia pestis* and *Rickettsia typhi* [8, 9]. The salivary glands of *X. cheopis* contain a variety of pharmacologically active proteins and peptides, including phosphatase family, esterase, apyrase, adenosine deaminase, mucins, antimicrobial peptide, antigen 5 family, FS or antigen 1 family, and some housekeeping proteins [10]. The mixture of these proteins and peptides can promote the blood-sucking behavior of *X. cheopis* and inhibit the host’s immune response. For example, thrombin inhibitors such as XC-42 and XC-43 are specific saliva inhibitors that could interfere with the coagulation ability of the host [11, 12]. In addition, XcAP-1, XcAP-2, and XcAP-3 in the saliva of *X. cheopis* have high affinity for biogenic amines and leukotrienes that play important roles in host hemostasis and inflammatory responses [13]. This means that these proteins may promote blood acquisition by sequestering agonists related to host’s hemostatic and immune responses. The FS family of peptides is a unique substance in fleas [14]. FS50 from the saliva of *X. cheopis* could block, which is a sodium channels and thus inhibit the activity of NaV1.5 channel [15]. Another saliva protein, FS48, can reduce T-cell activation by blocking Kv1.3 current and channel protein expression [16]. FS48 can also inhibit macrophage inflammation and downregulate the secretion of proinflammatory factors by inhibiting the Kv1.3 channel and subsequently inhibiting MAPK/NF- κ B signal pathway [17].

Some methods have been used to study host immune response to flea bites, e.g., flow cytometry, histology, and antibody detection, which revealed that flea bites only caused mild inflammatory reactions in mice, and continuous exposure to flea bites led to increased tolerance in the host without the occurrence of hypersensitivity reactions [18]. However, these methods may fail to capture the complex and deep-seated immune regulatory processes, and we have limited knowledge on the host immune response to flea bites and the corresponding transcriptome level changes of related genes.

Transcriptome sequencing can be used to detect transcriptional RNA molecules. By sequencing RNA molecules (RNA-seq), the composition and gene expression levels of transcripts can be quantified. RNA-seq has been widely used to study gene expression differences between individuals or the molecular mechanisms of related gene functions [19–22]. In this study, we perform RNA-seq on the spleen of mice to quantify the overall changes in gene expression of mice before and after flea bites, identify differentially expressed genes (DEGs), and analyze the functions and relationships of these genes. We attempt to elucidate detailed molecular mechanisms of the host response to flea bites, which may shed light on the impact of ectoparasites bites on the immune system of the host.

## Methods

### Animals and sample collection

A total of ten specific pathogen-free (SPF) male Kunming mice (8 weeks old) were purchased from Sibeifu (Beijing) Biotechnology Co., Ltd. These mice were randomly divided into two groups using a random number table method, with five mice in each group. One group was used as the control group (C1–C5), and the other group was used as the flea bite group (T1–T5). In the flea bite group, each mouse was exposed to 50 *X. cheopis* for 3 days. Then the mice were anesthetized and euthanized, and their spleens were quickly dissected for RNA extraction.

*X. cheopis* were initially collected from the *Rattus norvegicus* in the suburban district of Siping City, Jilin Province (Northeast China) in 2002, and were then introduced to our laboratory in 2003. The population of fleas has been maintained in laboratory conditions since then with the blood of SPF mice.

### RNA extraction

Total RNA was extracted from spleen samples of ten Kunming mice in the control group and flea bite group using TRIzol method. Nanodrop was used to measure the concentration of RNA, and 1% agarose gel electrophoresis was used to evaluate the degradation degree of RNA and check the potential contaminations. Agilent 2100 Bioanalyzer was used to accurately examine the integrity of RNA to achieve strict quality control of extracted RNA. Due to the low RNA integrity value of sample T1, we removed this sample from the experiment, and the remaining nine samples will be processed for further analysis.

### Library construction and quality control

The sequencing library was constructed as follows: MRNA with polyA tail was enriched by magnetic beads containing Oligo (dT), and the obtained mRNA was randomly broken into short fragments by divalent cations in fragmentation buffer. Using interrupted mRNA as a template and random oligonucleotides as primers, the first strand of cDNA was synthesized in the M-MuLV reverse transcriptase system. Subsequently, RNA strands were degraded by RNaseH, and the second strand of cDNA was synthesized by dNTPs as raw material in the DNA polymerase I system. After synthesizing cDNA strands, the cDNA was purified, and the purified double stranded cDNA was subjected to end repair, followed by adding an A-tail and connecting to a sequencing adapter. AMPure XP beads were then used to screen out cDNA with a length of approximately 370–420 bp. PCR amplification was performed, and the PCR product was purified again using AMPure XP beads, and ultimately the cDNA library was obtained.

Quality control was performed on constructed library. First, Qubit 2.0 Fluorometer was used to perform preliminary quantification on each library diluted to 1.5 ng/μL. Then Agilent 2100 Bioanalyzer was used to examine the insert size of the library, and real-time quantitative reverse transcription polymerase chain reaction (RT-qPCR) was used to accurately quantify the effective concentration of the library, which should be higher than 1.5 nM. The principle of RT-qPCR is to use fluorescent molecules as probes, which follow or bind to the target DNA or RNA molecules and emit a bright signal as the detection result. This technology determines the specific existence quantity of DNA or RNA by scanning each PCR cycle and the fluorescence signals in test tube.

### Transcriptome sequencing

After the quality control of the library, Illumina sequencing was performed according to the effective concentration above 1.5 nM and the target offline data volume of 6 GB, and 150 bp paired-end reads were generated.

### Bioinformatics analysis

#### Data processing and transcriptome assembly

After obtaining raw data in fastq format, we used fastp [23] software to filter out reads with sequencing joints or lower sequencing quality in the raw data. The criteria to filter out reads were: reads with adapters, reads that cannot determine base information, and reads with more than 50% bases having Phred score less than 5. HISAT [24] was used to compare clean reads with *Mus musculus* reference genome (MusMusculus.GCF_000001635.27) in NCBI. The genomic location of clean reads on the reference genome was obtained, and then the number of reads covered by each gene from start to end was counted. The featureCounts [25] was used to quantify the reads mapped to each gene. Finally, fragments per kilobase of exon model per million mapped fragments (FPKM) was used to correct for the effects of sequencing depth and gene length.

#### Analysis of differentially expressed genes (DEGs)

DESeq2 [26] was used to screen and analyze DEGs between the control group and flea bite group on the basis of the obtained gene expression levels. The multiple differences and significance of gene expression levels were calculated and *P*-value < 0.05 were selected as thresholds to screen for significant DEGs.

#### GO, KEGG enrichment, and PPI network analysis of DEGs

ClusterProfiler [27] was used to perform gene ontology (GO) and Kyoto Encyclopedia of Genes and Genomes (KEGG) pathway enrichment analysis of DEGs. *P* < 0.05 was used as the threshold for significant enrichment in GO and KEGG analysis. The rrvgo package in R language was used to simplify the GO enrichment analysis results [28]. The STRING database was used to analyze the protein–protein interaction (PPI) network of DEGs and the Cytoscape software was used to visualize the PPI network of DEGs.

#### Validation of RNA-sequencing (RNA-seq) DEGs through RT-qPCR analysis

To demonstrate the reproducibility and accuracy of RNA-seq gene expression data from Kunming mice spleen library, several genes were randomly selected from the screened DEGs and the reliability of transcriptome data was verified using RT-qPCR method. The reagent kit method was used to reverse transcribe RNA into cDNA. The RT-qPCR system (20 μL) was as follows: 2 × Taq Pro Universal SYBR qPCR Master Mix, 10 μL; upstream and downstream primers (10 μM), 0.4 μL; ddH2O, 7.2 μL; and cDNA template, 2 μL. The reaction conditions were as follows: 95 °C for 30 s, followed by 40 cycles of 95 °C for 10 s and 60 °C for 30 s; the collection of melting curve was used to the default process of the instrument. 2^−ΔΔCt^ method was used to determine the relative expression level of the selected gene. HRPT served as internal reference gene. Five samples from the control group and four samples from the flea bite group were tested, and each sample were tested three times. The gene information for real-time PCR was presented in Table S1.

## Results

### Evaluation of transcriptome sequencing data

The data obtained from sequencing were above 6.0 Gb for each sample. The quality scores of Q20 were higher than 96.48% for all samples and Q30 were higher than 91.38%. The GC content ranged between 48.83 and 50.14%. Approximately 92.93–94.56% of the clean reads were mapped to the reference genome of *Mus musculus* (ncbi_mus_musculus_gcf_000001635_27_grcm39), including 85.87 to 88.51% uniquely mapped reads. The summary of quality control for each sample were given in Table 1.

**Table 1:** Summary of sequence quality and mapped data of samples

Sample	Raw reads	Raw bases	Clean reads	Clean bases	Error rate (%)	Q20 (%)	Q30 (%)	GC content (%)	Total mapped	Multiple mapped	Uniquely mapped
C1	40682014	6.10G	40029902	6.00G	0.03	96.49	91.39	49.29	37388559 (93.40%)	2101763 (5.25%)	35286796 (88.15%)
C2	43927948	6.59G	42843854	6.43G	0.03	96.95	92.11	50.14	40512336 (94.56%)	2591031 (6.05%)	37921305 (88.51%)
C3	43464340	6.52G	42532386	6.38G	0.03	96.68	91.77	49.36	39524049 (92.93%)	2276082 (5.35%)	37247967 (87.58%)
C4	46702470	7.01G	45680468	6.85G	0.03	96.75	91.92	49.24	42833602 (93.77%)	2422880 (5.30%)	40410722 (88.46%)
C5	43363820	6.5G	42493876	6.37G	0.03	97.40	93.40	49.24	40145296 (94.47%)	3435981 (8.09%)	36709315 (86.39%)
T2	42048386	6.31G	41448078	6.22G	0.03	96.48	91.38	48.83	38820595 (93.66%)	2358650 (5.69%)	36461945 (87.97%)
T3	44253292	6.64G	43637396	6.55G	0.03	96.67	91.70	49.36	40810814 (93.52%)	2244332 (5.14%)	38566482 (88.38%)
T4	43391202	6.51G	42463748	6.37G	0.03	96.49	91.49	49.15	39541501 (93.12%)	3076598 (7.25%)	36464903 (85.87%)
T5	43665300	6.55G	42683166	6.40G	0.03	96.75	91.97	49.12	39820093 (93.29%)	2711386 (6.35%)	37108707 (86.94%)

### Differential gene expression analysis

**Fig. 1:**
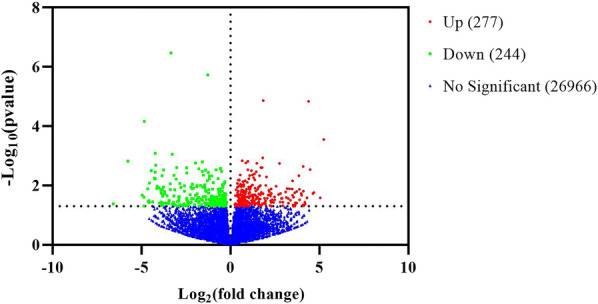
Volcano plot of global DEGs in the control group and the flea bite group. [Red dots represent significantly upregulated genes; green dots represent significantly downregulated genes; blue dots represent genes with insignificant differential expression. The *X*-axis represents the expression fold change of genes in two groups (|Log_2_fold change|> 0), and the *Y*-axis represents the significance level of gene expression differences between the two groups (*P* < 0.05)]

**Fig. 2:**
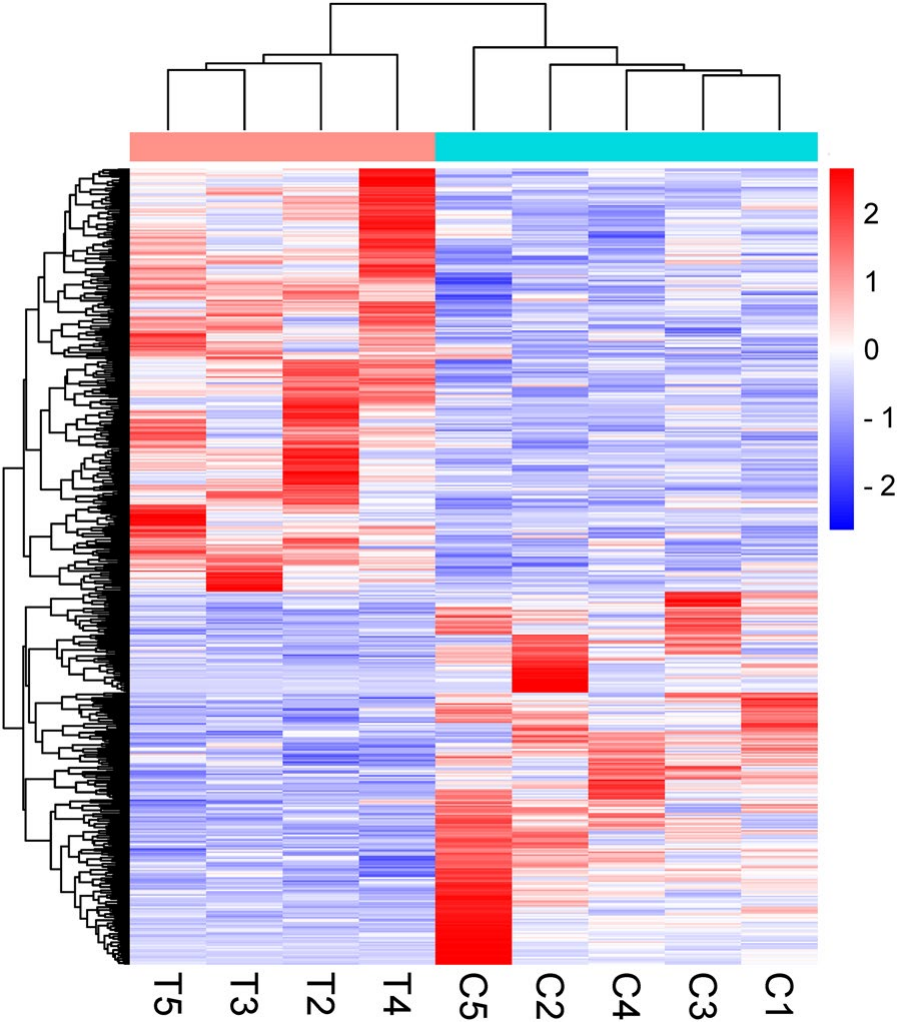
Expression profiles of DEGs in the control group and the flea bite group. (*X*-axis in figure represents the sample name, and *Y*-axis represents the normalized values of the FPKM of DEGs. Red represents upregulated genes, while blue represents downregulated genes)

We identified a total of 521 genes with significant differential expression between the control group and flea bite group (*P* < 0.05), with 277 genes upregulated and 244 genes downregulated (Fig. 1 and Table S2). We performed hierarchical clustering on the expression patterns of significant DEGs, which showed different expression patterns between the two groups. The samples of each group were clustered together by group, mainly manifested as two clusters (Fig. 2).

### Enrichment analysis of GO terms

**Fig. 3:**
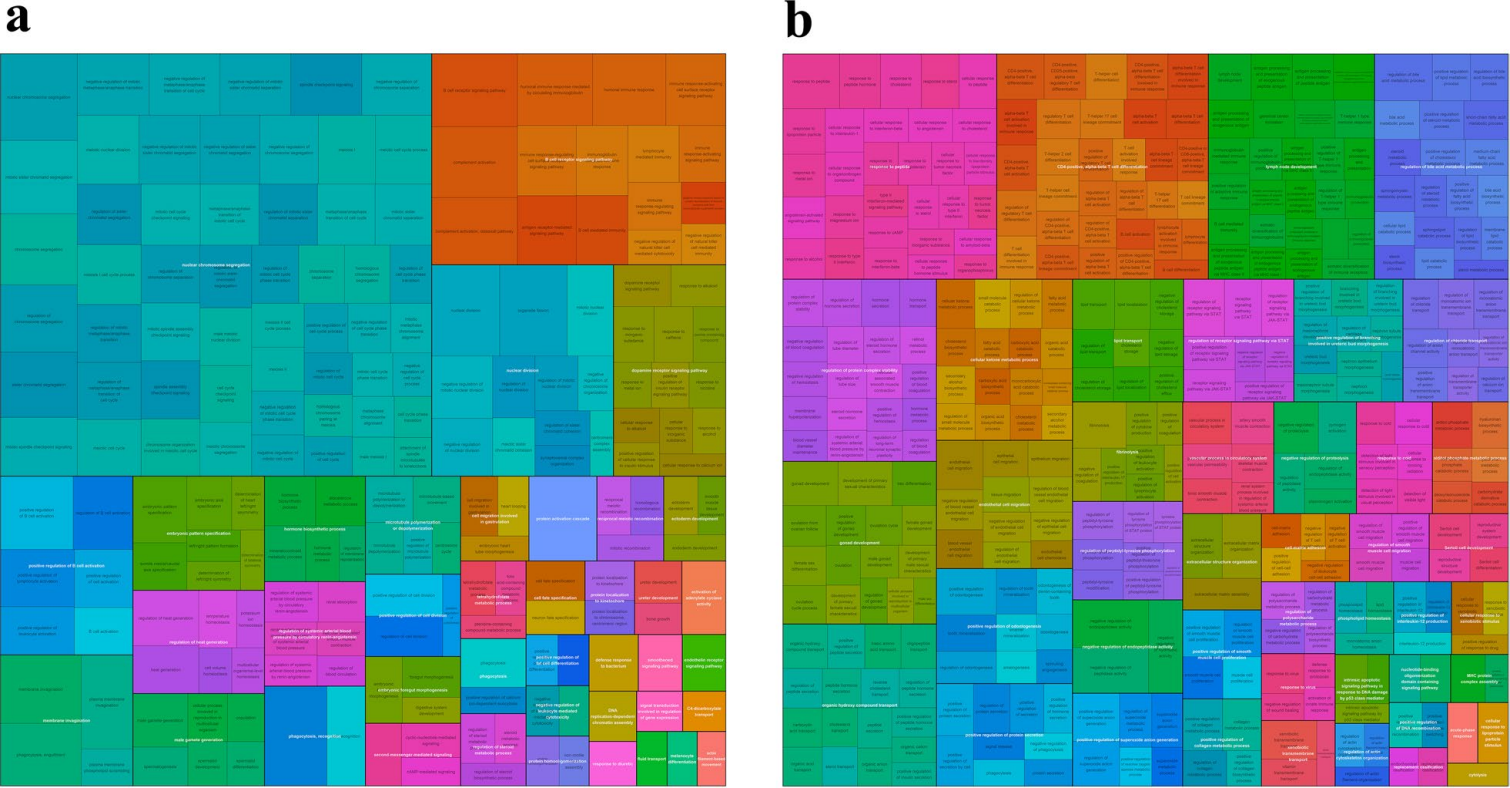
Tree diagram of GO term enrichment analysis based on semantic similarity clustering. (Simplify GO terms by grouping similar terms on the basis of semantic similarity, classify a large number of biological processes as GO terms, and make GO enrichment analysis results clearer and more concise. **a** Left figure shows the clustering of biological processes significantly enriched with upregulated DEGs. **b** Right figure shows the clustering of biological processes significantly enriched with downregulated DEGs)

GO enrichment analysis was performed on the DEGs. We identified 258 GO terms significantly enriched for the upregulated DEGs, including 196 terms of biological processes, 31 terms of cellular components, and 31 terms of molecular function. We identified 419 GO terms that were significantly enriched for the downregulated DEGs, including 360 terms of biological processes, 13 terms of cellular components, and 46 terms of molecular function. Most of the significant GO terms were biological processes, and we simplified these processes using R language based on the principle of semantic similarity (Fig. 3). There were 80 GO terms related to immune cells such as B cells and T cells, as well as immune regulatory processes, in both upregulated and downregulated DEGs.

### Enrichment analysis of KEGG pathway

**Fig. 4:**
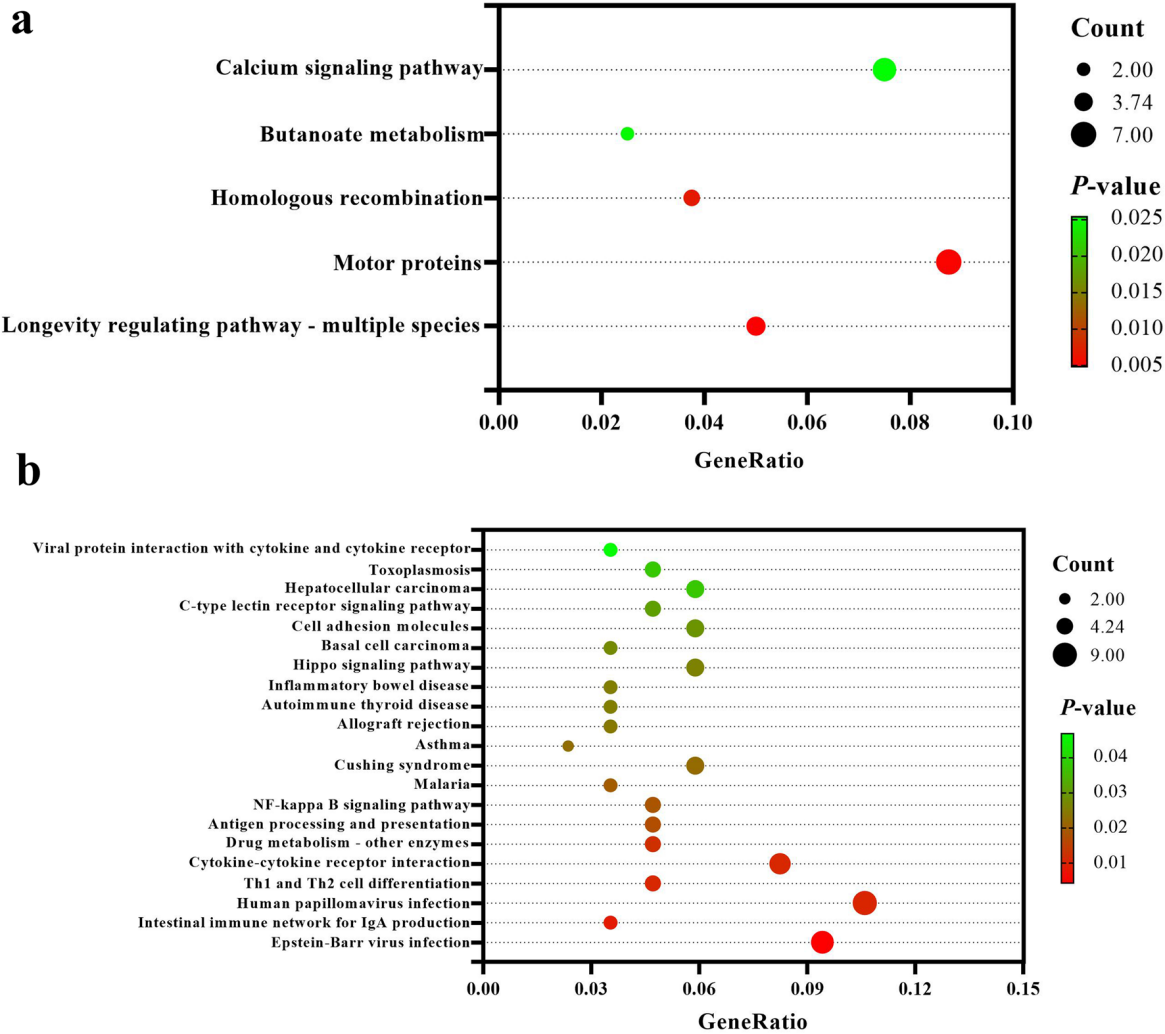
KEGG pathway enrichment analysis of DEGs. (The size of the dots represents the number of genes annotated in the KEGG pathway, and the color of the dots represents the significance of enrichment. **a** The above figure shows the KEGG pathway significantly enriched in upregulated DEGs. **b** The following figure shows the KEGG pathway significantly enriched in downregulated DEGs)

To gain a deeper understanding of the main metabolic and signaling pathways involved in DEGs between the two groups, we conducted KEGG pathway enrichment analysis. Through KEGG pathway enrichment analysis of DEGs, a total of 26 significantly enriched KEGG pathways were identified, including 5 pathways of upregulated DEGs and 21 pathways of downregulated DEGs. Multiple immune-related pathways were significantly enriched in downregulated DEGs, such as Th1 and Th2 cell differentiation, cytokine–cytokine receptor interaction, antigen processing and presentation, and NF-kappa B signaling pathway (Fig. 4).

### PPI network analysis of immune related DEGs

**Fig. 5:**
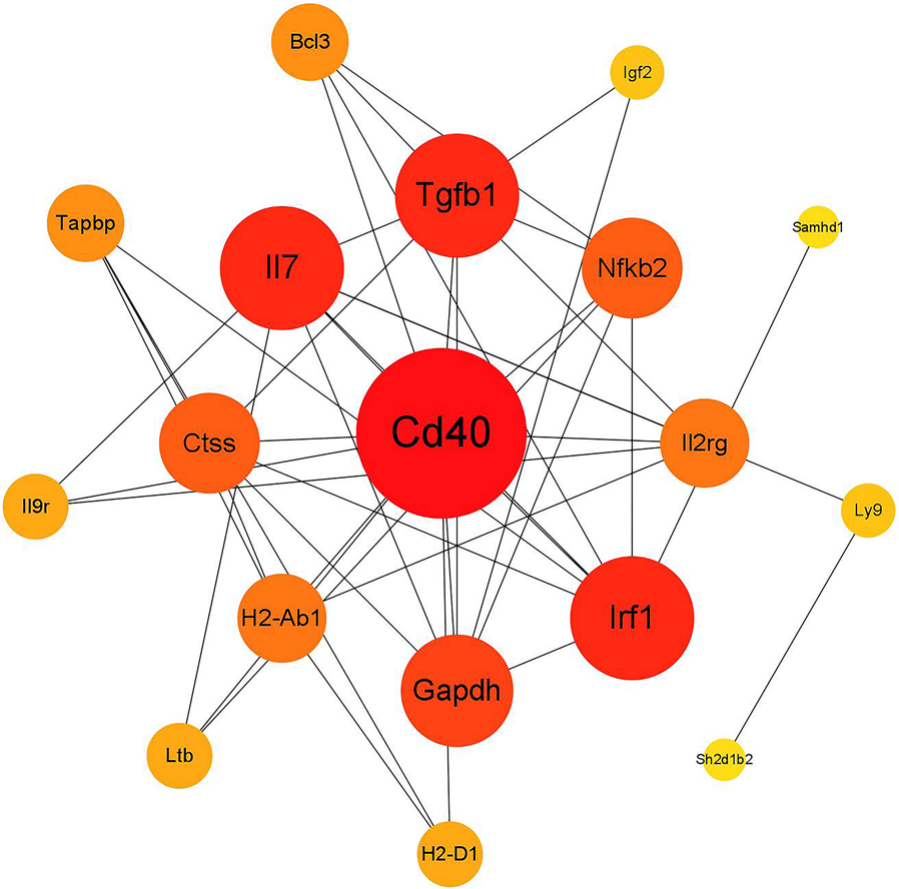
Protein–protein interaction network analysis of DEGs related to immunity

This study mainly focused on the changes in the immune system of the host after flea bites. Therefore, we summarized the DEGs involved in GO terms related to the differentiation and activation of immune cells such as B cells and T cells, as well as immune processes (immune-related DEGs could be found in Table S3). PPI network analysis was performed on them using the STRING database. We found that among these DEGs, there were 18 DEGs with 44 interaction relationships (Fig. 5). In this network, CD40 was a critical node that played an important role in maintaining tight connectivity throughout the entire network. The result suggested that after being bitten by fleas, the key node CD40 may be a highly central protein in the network and may play a core role in the immune regulation of the host.

### Validation of DEGs by RT-qPCR analysis

**Fig. 6:**
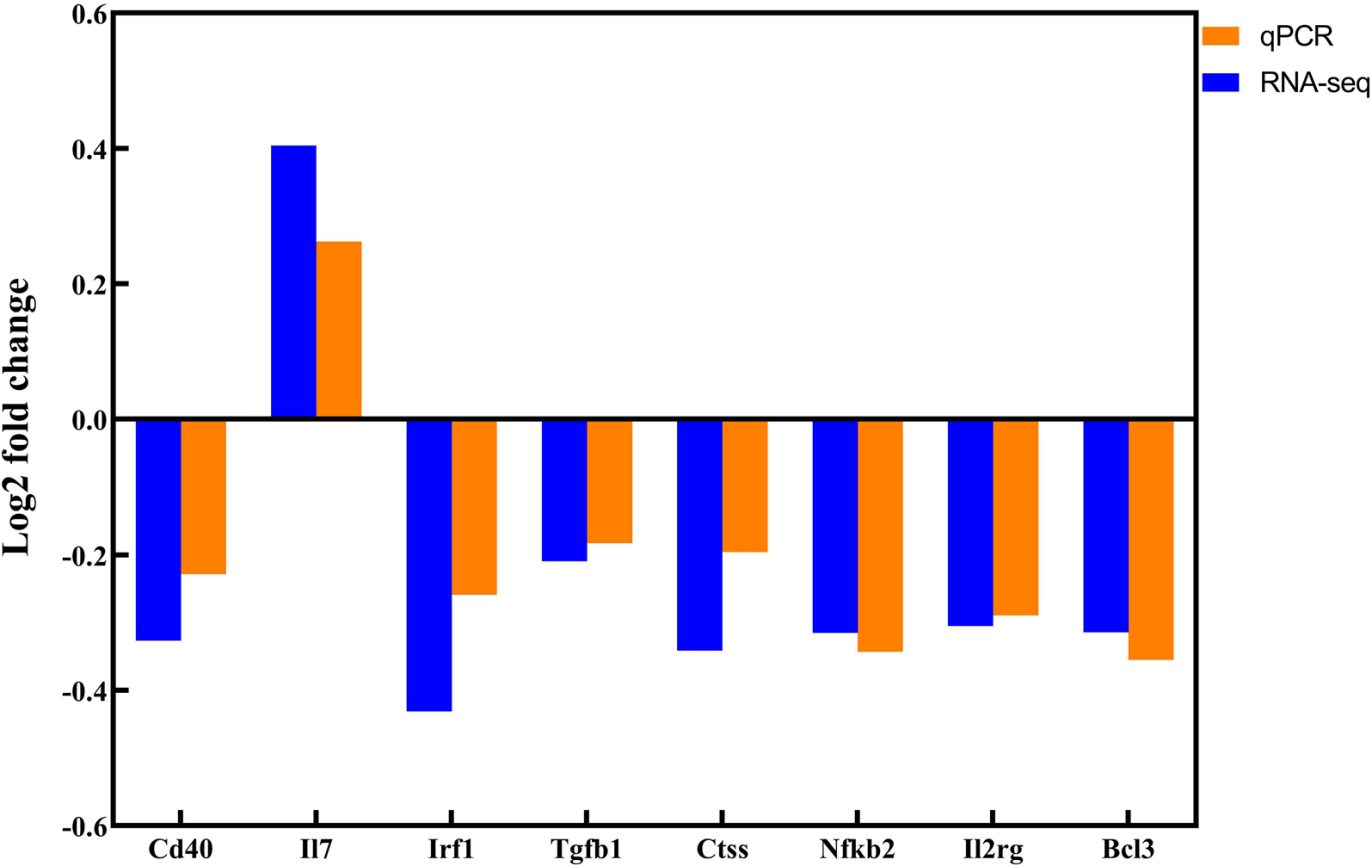
Validation of DEGs between the control group and the flea bite group by RT-qPCR. (*X*-axis represents DEG name, and *Y*-axis represents the log2 fold change)

To verify the RNA-seq expression results, we used RT-qPCR to evaluate the expression of randomly selected key DEGs obtained through PPI network analysis, including Cd40, Il7, Irf1, Tgfb1, Ctss, Nfkb2, Il2rg, and Bcl3. The results were basically consistent with those reflected by RNA-seq (Fig. 6), indicating the reliability of RNA-seq results.

## Discussion

After being bitten by *X. cheopis*, the cellular response of rats was almost entirely characterized by eosinophils. The skin response can be observed not limited to the bitten area, but also in other areas, despite being transient and extremely mild [29]. The results from a study on long-term exposure of mice to flea bites were consistent with this study. Regardless of the duration of exposure, the only skin sign observed in mice was a transient non-papular, non-edematous erythematous area, which may be due to blood leakage caused by the anticoagulant effect of flea saliva components. Flea bites on mice only caused mild inflammation [18]. After being bitten by *X. cheopis*, guinea pigs also exhibited a significant increase in eosinophils in their blood [30].

Through GO term enrichment analysis and clustering simplification based on semantic similarity, we found a total of 22 GO terms related to immune cell regulation and immune response among 277 upregulated DEGs, and 58 GO terms related to immune cell regulation and immune response among 244 downregulated DEGs. Then we conducted PPI network analysis on the DEGs involved in these GO terms and found that CD40 may act as a key node, interacting with many other immune-related proteins to form a dense network of interactions. This has important implications for us to understand the immune regulatory mechanism after flea bites.

CD40 belongs to the TNF receptor superfamily and is widely expressed in various types of cells, including B cells, dendritic cells, macrophages, monocytes and other immune cells, as well as epithelial cells, endothelial cells, fibroblasts, and other non-immune cells [31, 32]. When B cell is activated by antigens, the interaction between CD40 molecule expressed on its surface and CD40L molecule, which is the ligand of CD40 expressed by CD4 ^+^ T helper cells activated by antigens, is crucial for activating B cells and triggering humoral immune responses [33, 34]. Its ligand CD40L is also an important regulatory factor of the immune system, which has a strong effect on the activation of B and T cells, as well as on the development of effector functions in macrophages, B cells, and T cells [35]. The involvement of CD40 can also trigger the activation of epithelial cells, leading to the release of proinflammatory and antiinflammatory mediators, as well as molecules that promote fibrosis [36]. CD40/CD40L signaling enables cells in the blood to enhance the response of endothelial cells to inflammation and helps regulate hemostasis [37–39]. This may involve the activation and aggregation of platelets, or other physiological processes related to blood coagulation and hemostasis. Meanwhile, the interaction between CD40 and its ligand CD40L also plays an important role in hemostasis and coagulation. This study found that the expression of CD40 in Kunming mice was downregulated after flea bites. This may be because fleas, to facilitate their own blood sucking behavior, contain thrombin inhibitors such as XC-42 and XC-43 in their saliva, as well as proteins such as XcAP-1, XcAP-2, and XcAP-3, with high affinity for biogenic amines and leukotrienes that play important roles in the host’s hemostasis and inflammatory response, which slow down the coagulation and wound healing of the host [11–13]. We speculate that thrombin inhibitors may affect the host’s coagulation process, causing the blood in the bitten area to remain flowing for an extended period of time. The delay of this coagulation process may affect the signal transduction of the host’s immune system, thereby affecting the expression level of CD40 in the host's spleen. In addition, other proteins in flea saliva, such as XcAP-1, XcAP-2, and XcAP-3, may also be involved in regulating the host’s inflammatory response. These proteins may indirectly affect the expression level of CD40 in the spleen by regulating the host’s immune status. However, further experimental research is needed to verify these hypotheses.

Multiple studies have shown that CD40, as a cell surface receptor, plays a crucial role in parasitic immunity [40–43]. For example, CD40 may play a crucial role in activating macrophages in two different stages of cell-mediated immune response to Leishmania major infection, while the CD40/CD40L pathway plays an important role in regulating T cell and MΦ function in Leishmania infection hosts [44, 45]. After infection with *Toxoplasma gondii*, the expression of CD40 in the host is upregulated in both the acute and chronic stages of the disease [41]. The stimulation of CD40 in the body can reduce its own parasitic load, and blocking the interaction of endogenous CD40/CD40L will significantly increase its parasitic load [46]. This study found that after being bitten by fleas, the expression level of CD40 in Kunming mice was downregulated, and this molecule interacted with many other immune related proteins. This finding suggested that the CD40 of the host may play an important role in responding to immune challenges caused by flea bites. The downregulation of CD40 expression is likely due to a regulatory response of the host immune system to flea bites, which helps to balance the immune response and avoid excessive inflammation or immune overactivation. Because CD40 molecules play an important role in regulating B cells and T cells, the downregulation of CD40 may be able to maintain a balance of different cell types in the immune system, ensuring appropriate immune responses. Furthermore, pharmacologically active proteins and peptides in flea saliva may utilize the regulatory mechanisms of the host immune system to evade immune attacks, and downregulation of CD40 expression may involve immune escape. The downregulation of CD40 expression levels may also lead to impaired immune system function in the host, resulting in a decrease in its ability to respond to flea bites. Multiple studies have shown that the expression of CD40 in host cells decreased after saliva stimulation by ectoparasite [47–49].

When CD40 binds to its ligand CD40L, it also activates multiple signaling pathways, playing a crucial role in regulating the immune system and inflammatory response. The CD40/CD40L signaling pathway activates NF-κB, and MAPK and PI3K signaling pathways [50–52] regulate the activity and function of immune cells, affecting the occurrence and progression of immune and inflammatory responses. For example, NF-κB is an important transcription factor that plays a role in regulating the expression of inflammatory cytokines, including TNF-α, IL-1β and IL-6, among others [53–55]. CD40/CD40L binding activates the NF-κB signaling pathway, promoting the occurrence of inflammatory reactions and regulating the function of immune cells, which is of great significance in regulating the NF-κB signaling pathway [56–58]. The KEGG pathway enrichment analysis results showed that the downregulated DEGs of CD40 was enriched in multiple immune-related pathways, such as intestinal immune network for IgA production, cytokine–cytokine receptor interaction, NF-κB signaling pathway and cell adhesion molecules. In addition, among the downregulated DEGs related to immunity obtained through GO terms enrichment analysis and clustering simplification on the basis of semantic similarity, 11 DEGs were enriched in the KEGG pathway. The expression level of Il9r is most downregulated in the host after flea bites. The KEGG pathway enrichment analysis results indicated that both Il9r and CD40 were significantly enriched in the cytokine–cytokine receptor interaction pathway. There is literature supporting that the expression of Il9r on B cells is induced by CD40 stimulation, and Il9r signaling plays an important role in the differentiation of memory B cells and their humoral recall response [59]. After flea bites, the expression levels of CD40 and Il9r were both downregulated, which was likely to affect the activity of the immune cells and the effect of humoral immunity of the host, as well as reduce the ability of B cells to respond effectively to subsequent pathogen exposure. This may have an impact on the host’s ability to establish effective immune defenses to resist repeated challenges from ectoparasites, and further suggests that flea bites may affect the host’s immune response by regulating the expression of immune-related genes.

In summary, flea bites can stimulate a series of biological and immunological responses in mice. In-depth research on these biological and immunological responses could help to further understand the interaction between ectoparasites and hosts, as well as the mechanisms that may lead to disease transmission. The study aims to quantify transcriptional response of the host to flea bites in the short term. The changes in physiological parameters such as body weight and nutritional status were not considered, which may entail further studies.

## Conclusions

After 3-day exposure to *X. cheopis*, 277 upregulated DEGs and 244 downregulated DEGs were identified in Kunming mice, respectively. We found a total of 22 GO terms related to immune cell regulation and immune response among upregulated DEGs and 58 GO terms related to immune cell regulation and immune response among downregulated DEGs through clustering and simplification. Among the DEGs related to immune cell regulation and immune response, CD40 is an important gene that may play a core role in host immune regulation after flea bites. Our study suggests that flea bites could cause complex immune responses in the host, which expands our understanding of the response of mice to flea bites and sheds light on the interaction between ectoparasites and the host, as well as the adaptive response of the host to the pressure of ectoparasites.

### Supplementary Information

Supplementary Material 1.

Supplementary Material 2.

Supplementary Material 3.

## Data Availability

The raw data presented in the study are deposited in the NCBI repository, accession no. PRJNA1102695.
