# Supplementary material for: Transcriptome analysis of Kunming mice responses to the bite of Xenopsylla cheopis
Source: Parasit Vectors. 2024 Jun 7;17:250. doi: 10.1186/s13071-024-06331-4 (PMC11157846; doi:10.1186/s13071-024-06331-4)
Supplement: Supplementary file 1 — Supplementary Material 1. [file 13071_2024_6331_MOESM1_ESM.doc]

**Table S1 Primers for RT-qPCR**

| Name | Forward primer | Reverse primer |
| --- | --- | --- |
| HPRT | CAGTCCCAGCGTCGTGATTA | TGGCCTCCCATCTCCTTCAT |
| Cd40 | CTATGGGGCTGCTTGTTGAC | CTGGCTGGCACAAATCACAG |
| Il7 | CGATGAATTGGACAAAATGACAGG | TTGCGAGCAGCACGATTTAG |
| Irf1 | CCAGAGATTGACAGCCCTCG | TGCACAAGGAATGGCCTGAA |
| Tgfb1 | CTGATACGCCTGAGTGGCTG | TTTGGGGCTGATCCCGTTG |
| Ctss | GAGAGACCCTACCCTGGACT | CGTTCATGCCCACTTGGTAG |
| Nfkb2 | TGATGGCCCCTATCTGGTGA | ATAGGTCTTCCGGCCCTTCT |
| Il2rg | CGGTACACATTTCGGGTTCG | CAGCTTCCAGTGCAAACAAGG |
| Bcl3 | ATCCCCAGACCAAAGTGCCA | AGGAGCATCTTTCGGGGGAG |
