# Supplementary material for: Transcriptome analysis of Kunming mice responses to the bite of Xenopsylla cheopis
Source: Parasit Vectors. 2024 Jun 7;17:250. doi: 10.1186/s13071-024-06331-4 (PMC11157846; doi:10.1186/s13071-024-06331-4)
Supplement: Supplementary file 3 — Supplementary Material 3. [file 13071_2024_6331_MOESM3_ESM.doc]

**Table S3 Immune-related DEGs involved in GO terms**

| gene_id | the flea bite group | the control group | log2FoldChange | pvalue | gene_name | gene_length | gene_biotype | gene_description |
| --- | --- | --- | --- | --- | --- | --- | --- | --- |
| 19662 | 4.14 | 39.92 | -3.27 | 0.0009 | Rbp4 | 1297 | protein_coding | retinol binding protein 4%2C plasma%2C transcript variant 2 && sp|Q00724|RET4_MOUSE Retinol-binding protein 4 OS=Mus musculus OX=10090 GN=Rbp4 PE=1 SV=2 && PF00061:Lipocalin / cytosolic fatty-acid binding protein family |
| 14433 | 459.01 | 130.45 | 1.82 | 0.0012 | Gapdh | 1736 | protein_coding | glyceraldehyde-3-phosphate dehydrogenase%2C transcript variant X1 && sp|P16858|G3P_MOUSE Glyceraldehyde-3-phosphate dehydrogenase OS=Mus musculus OX=10090 GN=Gapdh PE=1 SV=2 && PF00044:Glyceraldehyde 3-phosphate dehydrogenase, NAD binding domain|PF02800:Glyceraldehyde 3-phosphate dehydrogenase, C-terminal domain |
| 629845 | 271.04 | 119.11 | 1.18 | 0.0032 | Ighv6-3 | 299 | V_segment | - && sp|P01801|HVM32_MOUSE Ig heavy chain V-III region J606 OS=Mus musculus OX=10090 PE=1 SV=1 && PF07686:Immunoglobulin V-set domain |
| 56045 | 6719.42 | 8354.98 | -0.31 | 0.0075 | Samhd1 | 4710 | protein_coding | SAM domain and HD domain%2C 1%2C transcript variant 2 && sp|Q60710|SAMH1_MOUSE Deoxynucleoside triphosphate triphosphohydrolase SAMHD1 OS=Mus musculus OX=10090 GN=Samhd1 PE=1 SV=3 && - |
| 625018 | 77.92 | 46.52 | 0.75 | 0.0092 | C4a | 5367 | transcribed_pseudogene | complement component 4A (Rodgers blood group)%2C transcript variant 1%2C non-coding && sp|P01029|CO4B_MOUSE Complement C4-B OS=Mus musculus OX=10090 GN=C4b PE=1 SV=3 && - |
| 23921 | 492.47 | 316.83 | 0.64 | 0.0096 | Sh2b2 | 3528 | protein_coding | SH2B adaptor protein 2%2C transcript variant X4 && sp|Q9JID9|SH2B2_MOUSE SH2B adapter protein 2 OS=Mus musculus OX=10090 GN=Sh2b2 PE=1 SV=2 && PF08916:Phenylalanine zipper|PF00017:SH2 domain|PF00169:PH domain |
| 15331 | 237.45 | 115.89 | 1.03 | 0.0112 | Hmgn2 | 1254 | protein_coding | high mobility group nucleosomal binding domain 2 && sp|Q86SG4|DPCA2_HUMAN Putative Dresden prostate carcinoma protein 2 OS=Homo sapiens OX=9606 GN=HMGN2P46 PE=5 SV=1 && - |
| 16362 | 3301.53 | 4452.20 | -0.43 | 0.0116 | Irf1 | 3359 | protein_coding | interferon regulatory factor 1%2C transcript variant 1 && sp|P15314|IRF1_MOUSE Interferon regulatory factor 1 OS=Mus musculus OX=10090 GN=Irf1 PE=1 SV=1 && PF00605:Interferon regulatory factor transcription factor |
| 14961 | 12311.64 | 18439.29 | -0.58 | 0.0129 | H2-Ab1 | 1193 | protein_coding | histocompatibility 2%2C class II antigen A%2C beta 1 && sp|P14483|HB2A_MOUSE H-2 class II histocompatibility antigen, A beta chain OS=Mus musculus OX=10090 GN=H2-Ab1 PE=1 SV=1 && PF00969:Class II histocompatibility antigen, beta domain|PF07654:Immunoglobulin C1-set domain |
| 16002 | 192.57 | 106.63 | 0.85 | 0.0161 | Igf2 | 5701 | protein_coding | insulin-like growth factor 2%2C transcript variant X3 && sp|P09535|IGF2_MOUSE Insulin-like growth factor II OS=Mus musculus OX=10090 GN=Igf2 PE=1 SV=1 && - |
| 668389 | 8.73 | 1.02 | 3.11 | 0.0164 | Ighv2-4 | 293 | V_segment | - && sp|P01821|HVM45_MOUSE Ig heavy chain V region MC101 OS=Mus musculus OX=10090 PE=1 SV=1 && - |
| 16196 | 420.07 | 317.55 | 0.40 | 0.0185 | Il7 | 3590 | protein_coding | interleukin 7%2C transcript variant 3 && sp|P10168|IL7_MOUSE Interleukin-7 OS=Mus musculus OX=10090 GN=Il7 PE=1 SV=1 && PF01415:Interleukin 7/9 family |
| 16199 | 53.51 | 207.62 | -1.96 | 0.0212 | Il9r | 7514 | protein_coding | interleukin 9 receptor%2C transcript variant X1 && sp|Q01114|IL9R_MOUSE Interleukin-9 receptor OS=Mus musculus OX=10090 GN=Il9r PE=2 SV=1 && - |
| 12051 | 1185.04 | 1474.06 | -0.31 | 0.0218 | Bcl3 | 1846 | protein_coding | B cell leukemia/lymphoma 3 && sp|Q9Z2F6|BCL3_MOUSE B-cell lymphoma 3 protein homolog OS=Mus musculus OX=10090 GN=Bcl3 PE=1 SV=2 && PF12796:Ankyrin repeats (3 copies) |
| 629906 | 119.52 | 43.83 | 1.44 | 0.0227 | Ighv1-42 | 310 | V_segment | - && sp|P01758|HVM14_MOUSE Ig heavy chain V region 108A OS=Mus musculus OX=10090 GN=Igh-VJ558 PE=4 SV=1 && PF07686:Immunoglobulin V-set domain |
| 626545 | 4.12 | 12.06 | -1.54 | 0.0237 | Trdv5 | 345 | V_segment | - && sp|A0JD37|TRDV3_HUMAN T cell receptor delta variable 3 OS=Homo sapiens OX=9606 GN=TRDV3 PE=1 SV=1 && PF07686:Immunoglobulin V-set domain |
| 628614 | 10.79 | 79.18 | -2.88 | 0.0240 | Ighv1-34 | 294 | V_segment | - && sp|P01758|HVM14_MOUSE Ig heavy chain V region 108A OS=Mus musculus OX=10090 GN=Igh-VJ558 PE=4 SV=1 && - |
| 380792 | 15.46 | 6.52 | 1.27 | 0.0243 | Ighe | 1480 | C_region | - && sp|P06336|IGHE_MOUSE Ig epsilon chain C region OS=Mus musculus OX=10090 PE=4 SV=2 && PF00047:Immunoglobulin domain|PF07654:Immunoglobulin C1-set domain |
| 12144 | 387.33 | 244.73 | 0.66 | 0.0252 | Blm | 6040 | protein_coding | Bloom syndrome%2C RecQ like helicase%2C transcript variant 1 && sp|O88700|BLM_MOUSE Bloom syndrome protein homolog OS=Mus musculus OX=10090 GN=Blm PE=1 SV=1 && PF16124:RecQ zinc-binding|PF00570:HRDC domain|PF00271:Helicase conserved C-terminal domain|PF08072:BDHCT (NUC031) domain|PF16202:N-terminal region of Bloom syndrome protein|PF00270:DEAD/DEAH box helicase|PF16204:BDHCT-box associated domain on Bloom syndrome protein|PF09382:RQC domain |
| 545378 | 4.41 | 0.56 | 2.90 | 0.0267 | Sh2d1b2 | 399 | protein_coding | SH2 domain containing 1B2 && sp|Q45HK4|SH21C_MOUSE SH2 domain-containing protein 1B2 OS=Mus musculus OX=10090 GN=Sh2d1b2 PE=1 SV=1 && PF00017:SH2 domain |
| 107817 | 622.54 | 785.33 | -0.33 | 0.0275 | Jmjd6 | 2789 | protein_coding | jumonji domain containing 6%2C transcript variant X1 && sp|Q9ERI5|JMJD6_MOUSE Bifunctional arginine demethylase and lysyl-hydroxylase JMJD6 OS=Mus musculus OX=10090 GN=Jmjd6 PE=1 SV=2 && - |
| 17085 | 1994.87 | 2487.24 | -0.32 | 0.0280 | Ly9 | 2591 | protein_coding | lymphocyte antigen 9%2C transcript variant 3 && sp|Q01965|LY9_MOUSE T-lymphocyte surface antigen Ly-9 OS=Mus musculus OX=10090 GN=Ly9 PE=1 SV=2 && PF13895:Immunoglobulin domain |
| 16994 | 7773.84 | 11612.87 | -0.58 | 0.0283 | Ltb | 1095 | protein_coding | lymphotoxin B && sp|P41155|TNFC_MOUSE Lymphotoxin-beta OS=Mus musculus OX=10090 GN=Ltb PE=2 SV=1 && PF00229:TNF(Tumour Necrosis Factor) family |
| 21939 | 1063.77 | 1334.35 | -0.33 | 0.0291 | Cd40 | 1879 | protein_coding | CD40 antigen%2C transcript variant 3 && sp|P27512|TNR5_MOUSE Tumor necrosis factor receptor superfamily member 5 OS=Mus musculus OX=10090 GN=Cd40 PE=1 SV=3 && PF00020:TNFR/NGFR cysteine-rich region |
| 18828 | 80.39 | 49.87 | 0.69 | 0.0329 | Plscr2 | 2117 | protein_coding | phospholipid scramblase 2%2C transcript variant A && sp|Q9DCW2|PLS2_MOUSE Phospholipid scramblase 2 OS=Mus musculus OX=10090 GN=Plscr2 PE=1 SV=3 && PF03803:Scramblase |
| 380824 | 16.36 | 52.79 | -1.69 | 0.0353 | Ighv1-66 | 294 | V_segment | - && sp|P06327|HVM52_MOUSE Ig heavy chain V region VH558 A1/A4 OS=Mus musculus OX=10090 GN=Gm5629 PE=2 SV=1 && - |
| 668589 | 296.12 | 42.82 | 2.79 | 0.0360 | Ighv1-80 | 294 | V_segment | - && sp|P01750|HVM06_MOUSE Ig heavy chain V region 102 OS=Mus musculus OX=10090 PE=1 SV=1 && - |
| 629930 | 6.16 | 0.79 | 2.95 | 0.0365 | Ighv8-6 | 301 | V_segment | - && sp|P01817|HV205_HUMAN Immunoglobulin heavy variable 2-5 OS=Homo sapiens OX=9606 GN=IGHV2-5 PE=1 SV=2 && PF07686:Immunoglobulin V-set domain |
| 21803 | 3854.51 | 4457.41 | -0.21 | 0.0366 | Tgfb1 | 2197 | protein_coding | transforming growth factor%2C beta 1 && sp|P04202|TGFB1_MOUSE Transforming growth factor beta-1 proprotein OS=Mus musculus OX=10090 GN=Tgfb1 PE=1 SV=1 && PF00019:Transforming growth factor beta like domain|PF00688:TGF-beta propeptide |
| 238427 | 133.58 | 52.11 | 1.36 | 0.0407 | Ighv6-6 | 300 | V_segment | - && sp|P01801|HVM32_MOUSE Ig heavy chain V-III region J606 OS=Mus musculus OX=10090 PE=1 SV=1 && PF07686:Immunoglobulin V-set domain |
| 16186 | 5241.61 | 6477.22 | -0.31 | 0.0415 | Il2rg | 1663 | protein_coding | interleukin 2 receptor%2C gamma chain%2C transcript variant a && sp|P34902|IL2RG_MOUSE Cytokine receptor common subunit gamma OS=Mus musculus OX=10090 GN=Il2rg PE=1 SV=1 && PF09240:Interleukin-6 receptor alpha chain, binding|PF00041:Fibronectin type III domain |
| 235320 | 66.47 | 39.69 | 0.74 | 0.0415 | Zbtb16 | 8201 | protein_coding | zinc finger and BTB domain containing 16%2C transcript variant X1 && sp|Q05516|ZBT16_HUMAN Zinc finger and BTB domain-containing protein 16 OS=Homo sapiens OX=9606 GN=ZBTB16 PE=1 SV=2 && - |
| 14964 | 28927.04 | 40738.00 | -0.49 | 0.0422 | H2-D1 | 1736 | protein_coding | histocompatibility 2%2C D region locus 1 && sp|P01899|HA11_MOUSE H-2 class I histocompatibility antigen, D-B alpha chain OS=Mus musculus OX=10090 GN=H2-D1 PE=1 SV=2 && PF07654:Immunoglobulin C1-set domain|PF00129:Class I Histocompatibility antigen, domains alpha 1 and 2 |
| 629842 | 27.14 | 12.37 | 1.12 | 0.0430 | Ighv13-2 | 300 | V_segment | - && sp|P01799|HVM30_MOUSE Ig heavy chain V-III region ABE-47N OS=Mus musculus OX=10090 PE=1 SV=1 && PF07686:Immunoglobulin V-set domain |
| 18034 | 2802.61 | 3487.82 | -0.32 | 0.0483 | Nfkb2 | 11300 | protein_coding | nuclear factor of kappa light polypeptide gene enhancer in B cells 2%2C p49/p100%2C transcript variant 1 && sp|Q9WTK5|NFKB2_MOUSE Nuclear factor NF-kappa-B p100 subunit OS=Mus musculus OX=10090 GN=Nfkb2 PE=1 SV=1 && - |
| 629860 | 39.08 | 19.09 | 1.03 | 0.0492 | Ighv1-12 | 294 | V_segment | - && sp|P01750|HVM06_MOUSE Ig heavy chain V region 102 OS=Mus musculus OX=10090 PE=1 SV=1 && - |
| 13040 | 6288.55 | 7968.52 | -0.34 | 0.0494 | Ctss | 1450 | protein_coding | cathepsin S%2C transcript variant 1 && sp|O70370|CATS_MOUSE Cathepsin S OS=Mus musculus OX=10090 GN=Ctss PE=1 SV=2 && PF00112:Papain family cysteine protease|PF08246:Cathepsin propeptide inhibitor domain (I29) |
| 780794 | 173.78 | 86.79 | 1.00 | 0.0494 | Ighv5-17 | 294 | V_segment | - && sp|P01763|HV348_HUMAN Immunoglobulin heavy variable 3-48 OS=Homo sapiens OX=9606 GN=IGHV3-48 PE=1 SV=2 && - |
| 21356 | 5693.99 | 7226.49 | -0.34 | 0.0499 | Tapbp | 2750 | protein_coding | TAP binding protein%2C transcript variant 1 && sp|Q9R233|TPSN_MOUSE Tapasin OS=Mus musculus OX=10090 GN=Tapbp PE=1 SV=2 && - |
